# Synergistic Enhancement of LiNO_3_-NaNO_3_-KNO_3_-NaNO_2_ Thermophysical Properties Through Dual Nano-Additives: SiO_2_ and MgO

**DOI:** 10.3390/nano15141094

**Published:** 2025-07-14

**Authors:** Chuang Zhu, Wenxuan He, Manting Gu, Dan Zhang, Baiyuan Tian

**Affiliations:** 1 School of Energy and Electrical Engineering, Qinghai University, Xining 810016, China; 220805040125@qhu.edu.cn (W.H.); ys240856010227@qhu.edu.cn (D.Z.);; 2 Engineer School, Qinghai Institute of Technology, Xining 810016, China

**Keywords:** nanocomposites, quaternary salt, nitrate, specific heat capacity, thermal diffusion coefficient

## Abstract

LiNO_3_-NaNO_3_-KNO_3_-NaNO_2_ has a relatively low phase-change temperature, making it suitable for low-temperature heat utilization systems. This study focuses on the performance optimization of the quaternary molten salt to advance its applicability. A series of nanocomposites consisting of nano-SiO_2_/MgO and the quaternary salt are prepared. Core thermophysical properties, including phase transition behaviors and thermal transport parameters, are quantified. The incorporation of nano-SiO_2_/MgO induces moderate adjustments to the melting point and latent heat yet demonstrates an obvious enhancement in specific heat capacity. Optimal doping at 0.7 wt.% SiO_2_ and 0.3 wt.% MgO yields a molten-state specific heat of 1.51 J/(g·K), representing a 6% increase over the undoped base salt (1.42 J/(g·K)). By combining the thermal diffusivity properties of the samples, this study found that the doping of nanoparticles typically induces new structures in molten salts that tend to enhance the specific heat capacity while simultaneously reducing thermal diffusivity.

## 1. Introduction

The profound transformation of the global energy structure and the introduction of green and low-carbon development goals have brought the development and utilization of clean energy to the forefront of international attention. Nevertheless, the intermittent and unstable characteristics of renewable energy sources like solar and wind power present significant challenges in the continuity and stability of energy supply. To overcome these challenges, energy storage technologies have become crucial in the efficient utilization of renewable energy. Thermal energy storage, using molten salt as a key material, offers advantages such as low vapor pressure, a high heat capacity, and strong stability [1,2]. It can be applied in various scenarios including solar thermal power generation, industrial waste heat recovery, crop environmental protection, and residential heating. The specific heat capacity is a crucial property of molten salts, closely linked to their heat storage density.

Scholars have widely recognized that incorporating nanoparticles into molten salts is a proven method of enhancing the specific heat capacity, based on extensive research over many years. Shin et al. [3] discovered that doping nano-SiO_2_ into metal chloride could increase its specific heat by 14.5% in their early research. Yu [4] discovered that the addition of Al_2_O_3_ nanoparticles or carbon nanotubes could significantly enhance the specific heat of ternary carbonate, with the maximum increase in specific heat reaching up to 41.2% (535–560 °C). Qiang Y et al. [5] found that when 0.1% SiO_2_ and 0.9% TiO_2_ nanoparticles were added to a mixed quaternary salt (Ca(NO_3_)_2_·4H_2_O–KNO_3_–NaNO_3_–NaNO_2_), the specific heat increased by 28.1% and the thermal conductivity improved by 53.7% compared to the mixed quaternary salt alone. Wei X et al. [6] observed that the addition of 2.5 wt.% 20 nm MgO nanoparticles to NaCl-CaCl_2_-MgCl_2_ increased the specific heat capacity of the base salt by 38.2%. Suraparaju K S et al. [7] found that dispersing hexagonal boron nitride (h-BN) nanoparticles at a concentration of 0.1 wt.% in HITEC molten salt increased the heat capacity by 27%, the latent heat by 72%, and the thermal stability by 7%. Seonjin J et al. [8] reported that at the optimal nanoparticle concentration (1 wt.%), the specific heat of KNO_3_-SiO_2_ nanofluid increased by 24.1% (solid state) and 28.1% (molten state), respectively. Mannan A M A et al. [9] demonstrated that adding 1 wt.% SiO_2_ nanoparticles to pure binary salt NaNO_3_-KNO_3_ and subjecting it to 3 h of ultrasonication increased the specific heat by 15.6%. Despite these notable research achievements by scholars, and their active discussions of, and investigations into, mechanisms for improving the thermophysical properties of molten salts, a widely accepted and relatively mature mechanism has yet to emerge. Currently, the more frequently discussed mechanisms include the semi-solid layer mechanism, the surface energy mechanism, and the interfacial thermal resistance mechanism, among others. As these mechanisms have been extensively debated over at least the past decade, it is unnecessary to elaborate on the details of each mechanism here.

This study focuses on a low-melting-point quaternary salt (LiNO_3_-NaNO_3_-KNO_3_-NaNO_2_) due to its significant potential applications in daily life [10]. The preparation of molten slat composites (MNs) from inorganic salts, nanoparticles of MgO, and SiO_2_ was conducted using a microwave muffle furnace, as microwaves facilitate the improvement of nanoparticle dispersion, leading to a reduction in nanoparticle agglomeration [11]. The rationale for selecting nanoparticles MgO and SiO_2_ as research objects lies in their low cost, significant application potential, widespread research focus, and the ease with which peer scholars can compare the findings. The concentration of nanoparticle additives was maintained at 1 wt.% throughout this study, predicated on the prevailing consensus within the existing study that this specific dosage typically enables a substantial enhancement in the specific heat capacity of composite materials.

The nanoparticles underwent characterization via SEM (surface morphology), TEM (particle size), and XRD (phase and crystallinity). The thermal energy storage properties of MNs were quantified using differential scanning calorimetry (DSC), while thermal transport parameters were determined through transient photothermal radiometry (the laser flash method). Finally, the potential mechanisms behind material performance changes were discussed through the analysis of the microstructures. This study implements a novel dual-additive strategy utilizing binary nanoparticle systems to analyze the specific thermophysical properties of molten salts, and the investigation specifically addresses the interplay between heat storage capacity and heat transfer characteristics. While contributing conceptual foundations to the mechanistic understanding of specific heat capacity enhancement, it must be acknowledged that the present work does not establish a comprehensive theoretical framework.

## 2. Experiments

### 2.1. Sample Preparation

The base salt was formulated as LiNO_3_ (17.50%)–NaNO_3_ (14.18%)–KNO_3_ (50.53%)–NaNO_2_ (17.78%) with a 94.3 °C melting point. All nitrate/nitrite precursors were procured from Aladdin Biochemical Technology (Shanghai, China), while nano-SiO_2_ (99.9% purity) and nano-MgO (99%) were obtained from Sinopharm Chemical Reagent Co. (Shanghai, China). The detailed characteristics of the nanoparticles are given in Table 1.

**Figure 1:**
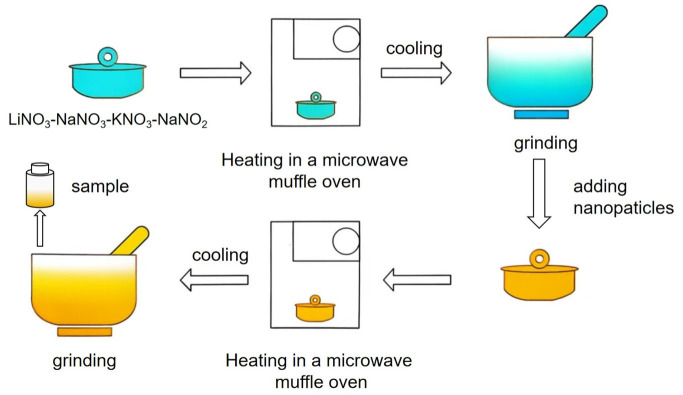
Synthesis pathway of MNs.

The four kinds of salts were uniformly mixed according to the specified ratios and then heated to 150 °C in a microwave muffle furnace, maintaining this temperature for 30 min to ensure complete melting. After the sample naturally cooled to room temperature, it was ground into powder to obtain the base salt. Based on this, MNs were prepared via a three-stage method: (1) the stoichiometric blending of additives and base salt (mass ratios detailed in Table 2) with mechanical homogenization via agate mortar milling; (2) thermal processing in a microwave-programmed muffle furnace under isothermal conditions (150 °C, 90 min) [11]; (3) post-synthesis processing involving cooling followed by particle size reduction through milling. This synthesis pathway is schematically characterized in Figure 1.

### 2.2. Measurement

#### 2.2.1. Heat Storage Performance

The specific heat measurements were conducted using a Synchronous Thermal Analyzer (STA-449F3, NETZSCH, Selb, BAV, Germany) with sealable aluminum crucibles.

The first step is baseline measurement. A crucible was placed in the sample and reference holders of the STA instrument, respectively, to initiate baseline acquisition. The temperature control program was set to execute the following sequence: (1) maintained isothermally at 200 °C for 10 min; (2) heated to 250 °C at a rate of 10 °C/min; (3) held isothermally for 15 min. Nitrogen served as both the protective gas (20 mL/min) and the purge gas (50 mL/min).

The second step is the sapphire reference measurement. A sapphire standard sample (diameter: 4 mm; mass: 12.58 mg) was placed in the sample aluminum crucible. Its DSC curve was measured using the baseline obtained in the previous step.

The third step is sample measurement. Following the sapphire reference measurement, the sapphire was removed from the sample aluminum crucible. A measured quantity of the sample was then loaded into the same crucible for DSC curve acquisition. The crucible was placed on the hot stage and held isothermally at 200 °C for 5 min and then subsequently weighed to obtain the dry sample mass. This value served as the actual sample mass input into the testing software (Table 3). The sample mass was deliberately kept small (≤10 mg) because larger quantities tend to adhere to the crucible walls and exhibit upward capillary migration during heating, thereby compromising measurement accuracy. The crucible lid was secured, and the assembly was pressurized under a sealing press to achieve hermetic closure. The sample was then analyzed using the baseline obtained in Step 1, yielding the DSC curve for the sample.

Finally, utilizing NETZSCH’s proprietary analysis software (Version 8.0.2), the specific heat capacity of the samples was determined by comparing the DSC curves of the sapphire standard and the samples. To ensure accuracy, all measurements were conducted in triplicate for each sample.

#### 2.2.2. Heat Transfer Properties

Thermal diffusivity was measured using a laser flash apparatus (LFA-467HT, NETZSCH, Selb, BAV, Germany). First, 0.25 g of the sample was weighed into a 12.7 mm diameter platinum-rhodium crucible. Then, the crucible was placed on a hot plate and heated until the sample melted. Subsequently, the crucible was capped, ensuring full contact between the lower surface of the lid and the molten sample surface.

The crucible was transferred to a room-temperature environment (23 ± 2 °C) and held statically for 5 min. Both the top and bottom surfaces of the crucible were then uniformly coated with graphite spray. After 3 min ambient drying, the crucible was loaded into the LFA-467HT sample holder for the diffusivity measurement.

Both the purge and protective gases were argon at 50 mL/min. The sample configuration was designated as a Three-layer structure in the instrument software, with Platinum-rhodium layers selected for both top and bottom encapsulation, sandwiching the Test sample layer (thickness: 0.49 mm) as the intermediate stratum. The sample coating option was designated as Graphite in the instrument software. The temperature program was configured to execute triplicate laser flashes at 10 °C intervals between 200 °C and 250 °C, with the analysis model explicitly selected as Standard. After the test was completed, the thermal diffusivity and standard deviation of these samples could be obtained by using the analysis software.

#### 2.2.3. Microstructure

The nanoparticle size distribution and crystallographic structure were characterized by TEM (Talos F200X, Thermo Fisher Scientific, Waltham, MA, USA) coupled with XRD (D/max2500PC, Rigaku, Tokyo, Japan). Microstructural features of both composite samples and pristine nanoparticles were investigated via FESEM (JSM-7900F, JEOL, Tokyo, Japan).

## 3. Results and Discussion

### 3.1. The Nanoparticles

**Figure 2:**
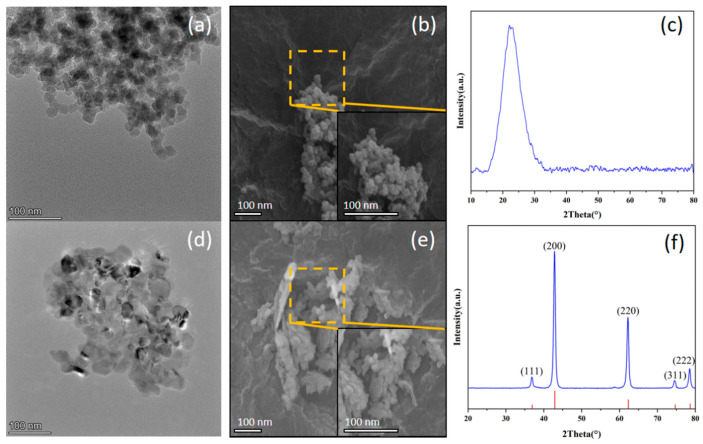
Characterizations of nano-SiO_2_ ((**a**) TEM, (**b**) SEM, (**c**) XRD) and nano-MgO ((**d**) TEM, (**e**) SEM, and (**f**) XRD).

Microstructural characterization was conducted on pristine nanoparticles (i.e., without salt incorporation). The nano-SiO_2_ nanoparticles exhibit a spherical morphology (about 30 nm diameter), as illustrated in Figure 2a,b. The XRD pattern obtained from the nano-SiO_2_ nanoparticle powder sample revealed an absence of distinct crystalline diffraction peaks, indicating its amorphous nature (Figure 2c). In contrast, nano-MgO particles demonstrate polyhedral crystallites (about 50 nm) as shown in Figure 2d,e. As can be seen from Figure 2f, the diffraction profile shows characteristic peaks at 36.94° (111), 42.92° (200), 62.30° (220), 74.69° (311), and 78.63° (222), exhibiting complete phase correspondence with standard MgO (JCPDS 45-0946). Clearly, these analyses demonstrate discernible structural differences between the two nanoparticle types.

### 3.2. Melting Point

**Figure 3:**
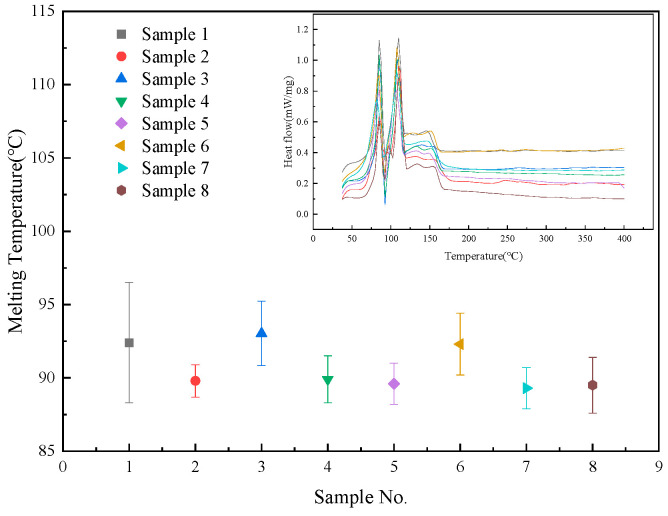
Melting temperatures and the corresponding heat flow curves.

**Figure 4:**
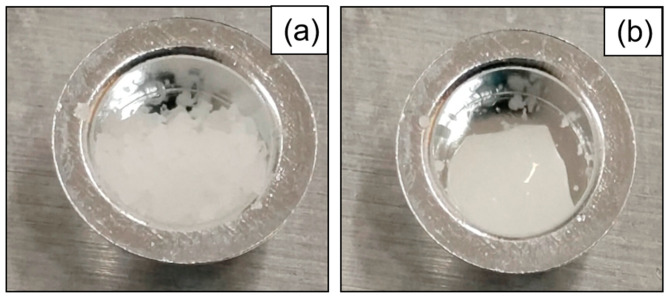
The states of the base salt at different temperatures: (**a**) 89 °C and (**b**) 150 °C.

The melting points and DSC curves of the samples are shown in Figure 3. It can be observed that all samples share a common feature in their DSC curves: the presence of two endothermic peaks at the same positions. The termination segment of the low-temperature peak and the initiation segment of the high-temperature peak overlap around 89 °C. To analyze the difference between the two endothermic peaks, the samples were held at 89 °C and 150 °C for 5 min on a hot plate, and their states were observed. Figure 4 illustrates the macroscopic state of the samples (using the base salt as an example). It is evident that the sample remained solid at 89 °C but became molten at 150 °C, indicating that the first endothermic peak corresponds to a solid–solid phase change, while the second peak represents a solid–liquid phase change. Therefore, in this study, the onset of the second peak is defined as the material’s melting point. The melting points of these samples primarily range between 90 °C and 95 °C, consistent with the melting point reported in another study (94.3 °C) [10].

The doping of nanoparticles had a slight impact on the melting points of the materials. Sample 3, which contained 0.9 wt.% MgO and 0.1 wt.% SiO_2_, shows a 0.6 °C rise in melting point. In contrast, the melting points of the other samples decreased to varying degrees. Sample 7 shows the lowest melting point at 89.6 °C. The reduction in the melting point of nano-molten salts is beneficial in expanding their application temperature range and can also lower the associated heating costs when used as thermal transfer materials. Additionally, doping with nanoparticles also induces variation in the latent heat of phase change. This phenomenon is attributable to increased lattice defects and the disordered state of molten salt at the nanoparticle surfaces [12,13,14].

### 3.3. Specific Heat Capacity

**Figure 5:**
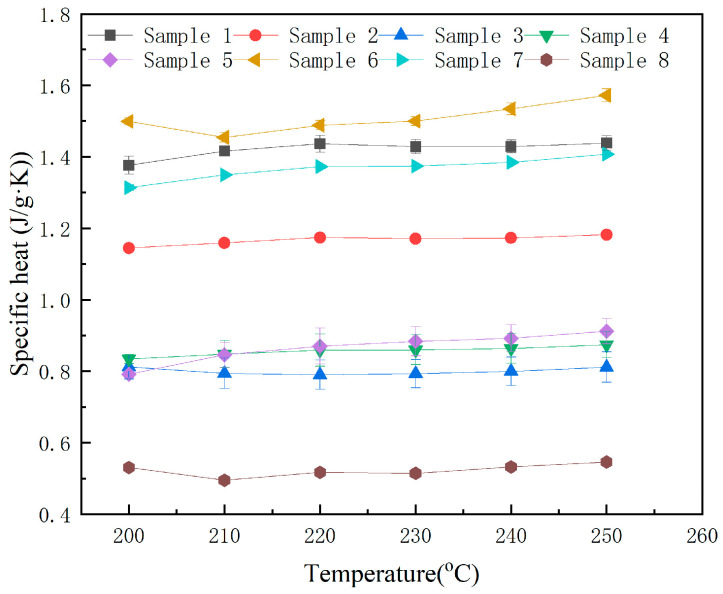
The specific heat of the samples at different temperatures.

The average specific heat capacity of the base salt in the molten state is 0.67 J/(g·K), as shown in Figure 5 and Table 4. The error bars in Figure 5 and the data in the parentheses in Table 4 represent the standard deviation. As evident from Figure 5 and Table 4, the small standard deviations across triplicate measurements indicate that most data points cluster closely around the mean values, demonstrating low variability in the results.

The results demonstrate that Sample 6 achieved a marginal enhancement in specific heat capacity, while all other samples exhibited varying degrees of reduction. The base salt maintained an average specific heat of 1.42 J/(g·K), whereas Sample 6 showed a 6% increase to 1.51 J/(g·K). Notably, Sample 8 displayed the lowest average specific heat at 0.52 J/(g·K). From the research results, it can be seen that the properties of the molten salt and the amount of nanoparticles added do not show a linear relationship. Such a phenomenon is very common in existing reports [15,16,17,18]. In fact, this is precisely a difficulty in the study of heat capacity enhancement mechanisms, reflecting the complexity of the influence of nanoparticles on the structure and properties of molten salts.

The increase in specific heat is related to a combination of multiple factors. Some studies suggest that nanoparticles possess a large specific surface area, which significantly enhances the interfacial thermal resistance, thereby improving the heat storage capacity of the composite material [3,17,19]. Other research indicates that the semi-solid layer structure formed between nanoparticles and molten salt is a contributing factor to the enhanced specific heat capacity of the composite material [3,14,19,20]. Another factor is the charged state of the nanoparticle surfaces, which can increase the specific heat capacity by enhancing the thermal expansion coefficient of ions [21]. The underlying reasons remain one of the current research hotspots.

### 3.4. Heat Transfer Characters

**Figure 6:**
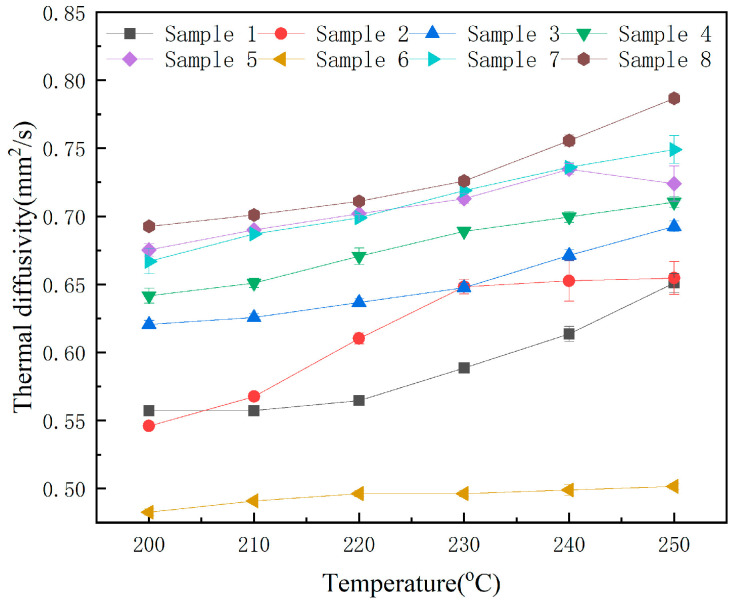
The thermal diffusion coefficient of the samples.

Temperature-dependent thermal diffusivity profiles of the samples across the operational temperature range (150–400 °C) are systematically characterized in Figure 6. The error bars in Figure 6 and the data in parentheses in Table 5 represent the standard deviation. From Figure 6 and Table 5, it can be seen that the standard deviation obtained from the three repeated measurements is relatively small, indicating that most data points are closely clustered around the average value, thereby showing a lower degree of variation in the results. With increasing temperature, the thermal diffusivity of most samples shows an upward trend (specific values can be found in Table 5), which is associated with particle movement at elevated temperatures. Elevated temperatures enhance ionic mobility within molten salts. This increased mobility, coupled with intensified interionic collisions, facilitates a more rapid process of heat transfer.

### 3.5. Discussion of the Relationship Between Specific Heat and Thermal Diffusivity

**Figure 7:**
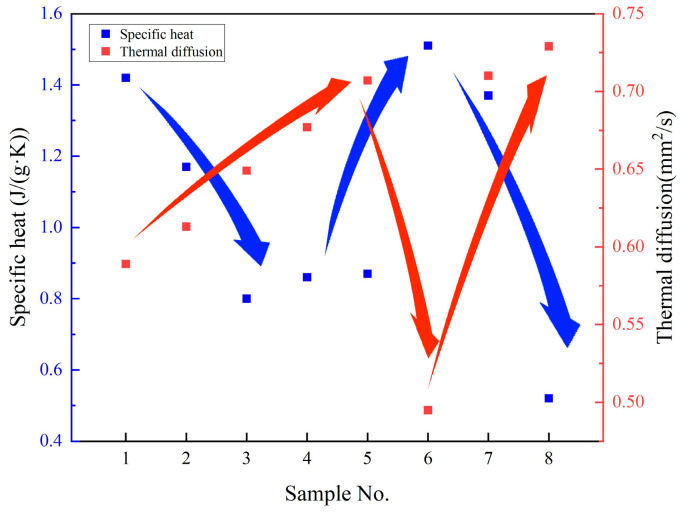
The relationship between specific heat and thermal diffusion coefficients.

Heat storage and heat transfer represent two critical thermophysical properties of molten salts. Accordingly, the mean specific heat capacity and mean thermal diffusivity across the temperature range of 200 °C to 250 °C for each sample are incorporated into Figure 7 for analysis. Thermal diffusivity is selected as the representative parameter for heat transfer characterization over thermal conductivity, owing to the latter’s multifactorial dependence on thermal diffusivity, density, and specific heat capacity. The data presented in Figure 7 consistently demonstrate an inverse relationship: samples exhibiting a lower specific heat capacity tend to possess higher thermal diffusivity, whereas those with a higher specific heat capacity generally display reduced thermal diffusivity. This is a newly identified phenomenon [22,23].

**Figure 8:**
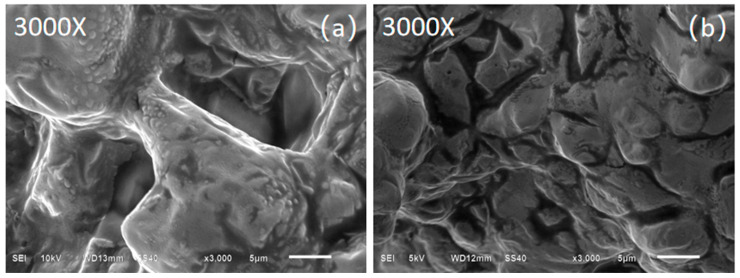
SEM image of (**a**) base salt and (**b**) 0.7%SiO_2_-0.3%MgO-base salt (Sample 6).

To elucidate the underlying mechanisms governing the observed thermal phenomenon, microstructural characterization was conducted via SEM analysis. In the base salt (Figure 8a), the inorganic salts are interconnected, and the surface is relatively smooth, which evidently facilitates heat transfer in the form of phonons throughout the material. For MNs, the microstructure is presented using Sample 6 as a representative case (Figure 8b). All other nanoparticle-doped samples exhibit comparable microstructural features. Microstructural analysis reveals that the nanoparticle-incorporated samples exhibit numerous interfacial boundaries. These interfaces constitute thermal barriers that impede heat diffusion processes. Conversely, established specific heat enhancement mechanisms indicate that the introduced interfaces impart additional interfacial thermal resistance—a recognized contributor to specific heat augmentation in nanocomposites. While the direct SEM characterization of materials in the molten state is experimentally infeasible, computational studies have confirmed the existence of semi-solid layers at nanoparticle interfaces [24]. These layers exhibit thermal transport properties functionally analogous to those of newly formed interfaces. We propose that this synergistic mechanism primarily underlies the observed variations in specific heat capacity and thermal diffusivity.

## 4. Conclusions

In this study, MNs were prepared as thermal energy storage materials using a quaternary salt mixture (LiNO_3_, NaNO_3_, KNO_3_, NaNO_2_) and two types of nanoparticles (SiO_2_, MgO) through the microwave method. The influence of the concentrations of the two nanoparticles on the heat transfer properties, thermal storage properties, and microstructure of the MNs was examined, and the main conclusions are as follows:

(1) The specific heat capacity measurement results of MNs exhibit significant nanoparticle-dependent variations. A 6% enhancement in molten-state specific heat capacity (1.51 J/(g·K)) is achieved at the optimal binary doping concentration of 0.7 wt.% SiO_2_ and 0.3 wt.% MgO, compared to 1.42 J/(g·K) for the base salt.
(2) The results obtained indicate that the combined doping of SiO_2_ and MgO in an appropriate ratio can enhance the thermal conductivity of the molten salt. The base salt exhibits an average thermal diffusion of 0.589 mm^2^/S. However, with the incorporation of 1.0 wt.% SiO_2_, this value increases significantly to 0.729 mm^2^/S, achieving a 24% enhancement compared to the pristine material.
(3) The dispersion of nanoparticles within molten salts introduces interfacial structures and/or semi-solid boundary layers. While these structures enhance the specific heat capacity, they simultaneously impede the thermal transport efficiency.

## Figures and Tables

**Table 1:** Parameters of nano-SiO_2_/MgO additives.

Characteristics	Parameters	
	Nano-SiO_2_	Nano-MgO
Size (nm)	About 30	About 50
Specific surface area (m^2^/g)	2.65	3.58
Density (g/cm^3^)	143	50
Thermal conductivity (W/(m·K))	8.3	36
Specific heat (J/(g·K))	0.99	0.92

**Table 2:** Mass ratio of salts to nano-additives in the samples.

Sample No.	LiNO_3_-NaNO_3_-KNO_3_-NaNO_2_	SiO_2_	MgO
1	1	0	0
2	0.99	0	0.01
3	0.99	0.001	0.009
4	0.99	0.003	0.007
5	0.99	0.005	0.005
6	0.99	0.007	0.003
7	0.99	0.009	0.001
8	0.99	0.01	0

**Table 3:** Sample mass for specific heat measurements.

Sample No.	1	2	3	4	5	6	7	8
Mass (mg)	5.61	5.33	9.67	5.06	5.36	5.28	5.05	5.36

**Table 4:** Specific heat values of the samples (J/g·K).

Sample No.	200 °C	210 °C	220 °C	230 °C	240 °C	250 °C
1	1.38 (±0.03)	1.42 (±0.01)	1.44 (±0.02)	1.43 (±0.02)	1.43 (±0.02)	1.44 (±0.02)
2	1.14 (±0.01)	1.16 (±0.00)	1.17 (±0.00)	1.17 (±0.00)	1.17 (±0.00)	1.18 (±0.01)
3	0.81 (±0.03)	0.79 (±0.04)	0.79 (±0.04)	0.79 (±0.04)	0.80 (±0.04)	0.81 (±0.04)
4	0.83 (±0.01)	0.85 (±0.04)	0.86 (±0.05)	0.86 (±0.04)	0.86 (±0.04)	0.87 (±0.04)
5	0.79 (±0.01)	0.85 (±0.03)	0.87 (±0.05)	0.88 (±0.04)	0.89 (±0.04)	0.91 (±0.04)
6	1.45 (±0.01)	1.45 (±0.01)	1.49 (±0.01)	1.50 (±0.01)	1.53 (±0.02)	1.57 (±0.02)
7	1.31 (±0.01)	1.35 (±0.00)	1.37 (±0.00)	1.37 (±0.00)	1.38 (±0.00)	1.41 (±0.00)
8	0.53 (±0.01)	0.50 (±0.01)	0.52 (±0.00)	0.51 (±0.01)	0.53 (±0.00)	0.55 (±0.00)

**Table 5:** Thermal diffusion coefficient values of the samples (mm^2^/S).

Sample No.	200 °C	210 °C	220 °C	230 °C	240 °C	250 °C
1	0.557 (±0.003)	0.557 (±0.003)	0.565 (±0.003)	0.589 (±0.003)	0.614 (±0.006)	0.651 (±0.007)
2	0.546 (±0.000)	0.568 (±0.003)	0.610 (±0.004)	0.648 (±0.006)	0.653 (±0.015)	0.655 (±0.012)
3	0.621 (±0.003)	0.626 (±0.003)	0.637 (±0.003)	0.648 (±0.003)	0.671 (±0.004)	0.693 (±0.004)
4	0.642 (±0.006)	0.651 (±0.003)	0.671 (±0.006)	0.689 (±0.000)	0.700 (±0.004)	0.710 (±0.004)
5	0.675 (±0.005)	0.690 (±0.00)	0.702 (±0.001)	0.713 (±0.003)	0.735 (±0.004)	0.724 (±0.013)
6	0.483 (±0.002)	0.491 (±0.000)	0.496 (±0.002)	0.496 (±0.002)	0.499 (±0.004)	0.502 (±0.002)
7	0.667 (±0.001)	0.687 (±0.000)	0.699 (±0.000)	0.719 (±0.000)	0.736 (±0.003)	0.749 (±0.010)
8	0.693 (±0.000)	0.701 (±0.003)	0.711 (±0.000)	0.726 (±0.003)	0.756 (±0.004)	0.787 (±0.004)

## Data Availability

The data presented in this study are available on request from the corresponding author.
